# Plasma Zinc Levels in Patients with Chronic Leg Ulcers at University College Hospital, Ibadan, Nigeria

**DOI:** 10.29252/wjps.10.2.40

**Published:** 2021-05

**Authors:** Adedoyin Babatunde Ojo, Odunayo Moronfoluwa Oluwatosin, Samuel Adesina Ademola, Olubayo Michael Akinosun

**Affiliations:** 1 Department of Surgery, University College Hospital, Ibadan, Nigeria;; 2 Department of Surgery, College of Medicine, University of Ibadan, Nigeria;; 3 Department of Chemical Pathology, University College Hospital, Ibadan, Nigeria

**Keywords:** Zinc, Chronic leg ulcers, Nigeria

## Abstract

**BACKGROUND:**

Chronic leg ulcers are defects in the skin below the level of knee persisting for more than six weeks and shows no tendency to heal after three or more months. Zinc is a necessary component of several DNA & RNA polymerases and transferases essential for cell proliferation. Zinc deficiency is known to retard wound healing by preventing cellular mitosis and disturbance of fibroblast function and collagen synthesis. This study compares zinc levels in patients with chronic leg ulcers with apparently healthy individuals.

**METHODS:**

Five milliliters of venous blood samples was taken from twelve patients with chronic leg ulcers. Five milliliters of venous blood samples was also taken from a control group, who do not have chronic leg ulcers, but are matched with the cases in age (± 5 yr), sex, socioeconomic status and body mass index (± 3 kg/m^2^). Plasma zinc levels was determined by atomic absorption spectrophotometry. Principal exposure include the socio-demographic characteristics of patients, duration of symptoms before presentation, signs of ulcer–site, number, size, depth, base, edge, presence of discharge, enlarged lymph nodes and local neurovascular integrity. The outcome variables include plasma levels of biochemical markers measured –Zinc, serum albumin, total protein.

**RESULTS:**

The plasma zinc levels was significantly lower (*P*<0.01) in patients with chronic leg ulcer (92.808±16.689 µg/dl) as compared with control subjects (109.413 ± 9.304 µg/dl). There was no statistical difference in albumin and protein levels in both groups.

**CONCLUSION:**

Patients with chronic leg ulcers have significantly lower plasma zinc levels than apparently healthy individuals.

## INTRODUCTION

An ulcer is the loss of continuity of the surface epithelium. The underlying tissues may be similarly affected. There are several causes of an ulcer, but necrosis of cells is the immediate cause^1^. 

Chronic leg ulcers are defects in the skin below the level of knee persisting for more than six weeks and shows no tendency to heal after three or more months^2^.

Venous disease is responsible for between 60%–70% of all ulcers in the lower leg in countries such as England and Australia^3^^,^^4^. Other causes include arterial ischaemic ulcers, haematological disorders, infective diseases, metabolic disorders, traumatic ulcers, neuropathic ulcers and neoplastic ulcers^5^^,^^6^.

The mechanism for venous ulcer development has not been fully established. The only accepted cause of ulceration at present is ambulatory venous hypertension. The venous hypertension may be as result of incompetence of saphenous vein valves, perforating veins or deep veins^3^. Other proposed hypotheses include static blood within superficial veins leading to hypoxia and tissue death^7^. Another is the concept of white cell trapping in that leucocytes were found to decrease in the venous effluent coming out of dependent limbs. Leucocyte trapping and activation in the microcirculation results in lipodermatosclerosis and ulceration in patients with chronic venous disease^8^. However, this has not been confirmed by further investigations^3^.

On the microvascular level, there is alteration and distention of the dermal capillaries with leakage of fibrinogen into the tissues; polymerisation of fibrinogen into fibrin cuffs leads to perivascular cuffing that can impede oxygen exchange and contribute to ulceration^2^. Reactive oxygen species and proteolytic enzymes are increased in ulcer environment leading to tissue damage. Inhibition of growth factors and abnormal fibroblasts may contribute to development of ulcer^9^.

Repeated trauma, poor perfusion or oxygenation, and excessive inflammation contribute to causation and perpetuation of chronicity of ulcers. Unresponsiveness to normal regulatory signals has also been implicated as a predictive factor of chronic ulcers. The clinically characteristic picture is that of an ulcer that fails to re-epithelialise despite the presence of adequate granulation tissue^2^.

Zinc deficiency, uncommon except in children in Middle East, is known to retard wound healing by preventing cellular mitosis and disturbance of fibroblast function and collagen synthesis^10^. The optimal range of plasma zinc is 90–150 µg/dl^11^. Low plasma zinc level is defined as ≤ 65 µg/dl^12^^-^^14^.

Zinc is a necessary component of several DNA & RNA polymerases and transferases essential for cell proliferation^10^. Zinc supplementation may improve wound healing. However, this needs to be cautiously done as excessive zinc levels may hinder macrophage migration and phagocytosis and thus impair wound healing^10^. The recommended daily zinc intake for an adult ranges from 8–11 mg. The Food Standards Agency and the Department of Health in the UK advise that intake of zinc should not exceed 25mg/day. Ingesting more than 200 mg/day or a prolonged intake of 50-150 mg/day can result in toxicity^15^.

We aimed to compare zinc levels in patients with chronic leg ulcers with apparently healthy individuals.

## MATERIALS AND METHODS

This study was conducted in the Department of Plastic, Reconstructive and Aesthetic Surgery, University College Hospital (UCH) Ibadan, Nigeria. The University College Hospital, Ibadan is an 850 bedded Federal Government owned teaching hospital located along Queen Elizabeth road, Ibadan, Oyo State in South Western Nigeria. The location of the hospital is strategic as it serves Ibadan metropolis with referrals from all over Nigeria and West Africa. The Department of Plastic, Reconstructive and Aesthetic Surgery is one of the 66 departments in the hospital.

Ethical approval was obtained from the joint University of Ibadan/University College Hospital Ethical Review Committee and the declaration of Helsinki was adopted. Informed consent for participation in this study was obtained. The procedure was explained to the participants and basic required information such as socio-demographic parameters was obtained. The informed consent form was also translated to local language (Yoruba) for easy communication.

Twelve consecutive patients with chronic leg ulcers presenting at the Surgical Outpatient (SOP) clinic of Department of Plastic, Reconstructive and Aesthetic Surgery were prospectively enrolled at University College Hospital, Ibadan over the study period. Twelve patients without chronic leg ulcers matched with the cases in age (± 5 yr), sex, socio-economic status and body mass index (±3 kg/m^2^) served as controls.

Individuals on nutritional supplementation containing zinc, individuals with recurrent chronic leg ulcers who have had a previous medical intervention and individuals with co-morbid conditions such as diabetes, haemoglobinopathy, retroviral disease were excluded from the study.

Five milliliters of venous blood samples was taken from the patients on one occasion. The blood samples were collected in both plain and heparinised bottles, and centrifuged immediately. The supernatant plasma and serum were separated and frozen at -40 ^o^C until analysis. Plasma zinc levels was measured with atomic absorption spectrophotometry. It was performed on a Beck 200 (AAS). Serum protein and albumin levels were measured using Biuret and Bromcresol green methods respectively. Lower detection limits used were 1.0g/L and 0.5g/L respectively^16^. 

Plasma zinc, serum protein and albumin were assayed by one laboratory scientist. Quality control samples were included. The Scientist was not aware of the patient’s characteristics in order to minimise assessment bias in the study.

The socio-economic class of the participants was determined using the common three-stratum model – the wealthy and powerful upper class that own and control the means of production; middle class of professional workers, small business owners and low-level managers; and a lower class who rely on low-paying wage jobs for livelihood. The average monthly income of the participants was used to keep these classes in perspective.

A detailed clinical examination was done involving a general examination, examination of the ulcer(s) and also vital signs measurement. Data was collected and entered into a proforma used for analysis. Explanatory variables include the Socio-demographic characteristics of patients, age at presentation, duration of symptoms before presentation; other symptoms and signs such as fever, lethargy and dyspnea, and clinical description of the ulcer.

The outcome variables include plasma levels of the biomarkers – zinc, total protein and albumin.

Statistical analysis was carried out using SPSS ver. 22 (Chicago, IL, USA) and descriptive data was represented in tables. Test for statistical association for the outcome variables (such as serum levels of bio markers) was done using correlation and regression analysis, Paired Students t-test, and Pearson correlation coefficient. The level of significance was set at *P* of 0.05.

## Results

The study comprised of 12 patients with chronic leg ulcers. Twelve patients without chronic leg ulcers matched with the cases in age (± 5 yr), sex, socio-economic status and body mass index (± 3kg/m^2^) were used as controls to eliminate confounding factors.

Out of the 12 patients enrolled in this study as cases, 4 (33.3%) were female while 8 (66.7%) were males. The age range of the cases was 22 – 75 yr while that of the controls was 26–71 yr. Concerning the ethnicity of the patients, 20 (83.3%) were Yoruba, 2 (8.3%) were Igbo and 2 (8.3%) were Urhobo. Seven (58.3%) of the cases were of a low socio-economic class while 5 (41.7%) were of middle socio-economic class. The duration of ulcer in the 12 cases ranged between 2 months and 120 months with an average of 6.5 months. Eight (66.7%) of the cases had one ulcer while four (33.3%) had multiple ulcers. The mean plasma zinc was significantly lower (*P*<0.01) in patients with chronic leg ulcers (92.808±16.689 µg/dl) compared with the controls (109.413±9.304 µg/dl).

The difference in the mean albumin and protein levels of the patients with chronic leg ulcers and the controls was not statistically significant (*P*>0.2). Using Pearson correlation coefficient, plasma zinc levels had an inverse correlation with the duration of ulcer in patients with chronic leg ulcers. Using multivariate analysis, zinc levels for patients in low and middle socioeconomic classes were 103.125 ± 5.644 µg/dl and 113.987 ± 7.126 µg/dl respectively (*P*=0.45) (Table 1). 

**Table 1:** Characteristics of study subjects

**Parameter**	**Patient (n=12)**	**Controls (n=12)**	
Age range (yr)	22–75	26–71	
Duration of ulcer (months)	2–120	-	
Number of ulcer	One ulcer 8 (66.7%) Two ulcers 4 (33.3%)	-	
Zinc (µg/dl)	92.808 ± 16.689	109.413 ± 9.304	*P*<0.01
Albumin (g/dl)	3.87 ± 0.50	3.48 ± 0.60	*P*>0.2
Protein (g/dl)	7.97 ± 0.84	7.05 ± 1.06	*P*>0.2

## DISCUSSION

The pathogenesis of chronic leg ulcer is multifactorial and the physiological role of zinc has been implicated in wound healing. Wound healing is a complex process involving the stages of inflammation, proliferation and maturation that occur on a continuum from injury to healing^17^. For optimum wound healing, these stages must be progressed through smoothly and efficiently; and zinc has an identifiable role in all three stages. The role of zinc in wound healing is multifactorial. It is required for collagen synthesis, cell proliferation, and immune function. All of these are essential for tissue regeneration and repair^18^.

The common three-stratum model of socio-economic class was used –the wealthy and powerful upper class that owns and controls the means of production; middle class of professional workers, small business owners and low-level managers; and a lower class who rely on low-paying wage jobs for livelihood. A low socio-economic class has been found to increase the risk of zinc deficiency^19^.

Patients with known chronic illnesses were excluded from both cases and control as some chronic illnesses such as diabetes are associated with low zinc levels. Olaniyan et al studied serum copper and zinc levels in Nigerian type 2 diabetic patients in 2012 and found that the mean serum zinc was significantly lower in diabetic patients compared with healthy subjects (*P*<0.001)^20^.

The gender distribution of the patients showed a male preponderance, a finding similar to earlier report of Iyun et al^21^. The Male to Female ratio in this study was 2 to 1, while that for the study by Iyun et al was 1.6 to 1. However, the gender distribution of this study should be interpreted with caution considering the fact that a consecutive sampling was employed and this sampling method is prone to selection bias.

The mean plasma levels of zinc was significantly lower in patients with chronic leg ulcers as compared to the controls (*P*<0.01). This is similar to results gotten from another study in which seventeen patients with chronic leg ulcers (age range 47–90) were studied. Comparison of nutritional serum indices between patients with chronic leg ulcers and an age matched control population was done. Zinc and other micronutrients were screened on fasting blood samples and study data were compared with data obtained from the nutritional status survey (NSS). Significant lower levels of zinc (*P*<0.0001 men, *P*=0.027 women) were found in chronic ulcer patients^22^.

However, there was no significant difference between the albumin and protein levels in both groups in this study. There was also no correlation of albumin and protein levels with duration of ulcers in patients who had chronic leg ulcers. This is in contrast to another study in which lower serum albumin and protein levels were significantly associated with chronic leg ulcers^23^. In a study in which strength of association between chronic skin ulcers and albumin levels was compared, they found out that patients with poor outcomes had lower albumin levels (*P*<0.01)^24^. Moreover, on multivariate analysis, albumin levels (0.20 per unit, 0.07-0.60) remained a significant independent predictor of poor wound healing outcomes. 

The reason for similar albumin and protein levels in this study for both groups could be as a result of exclusion of chronic illnesses in the cases and controls. Other studies that identified lower serum albumin and protein levels in patients with chronic leg ulcers, these patients also had other chronic illnesses such as sickle cell disease or diabetes mellitus.

Using Pearson correlation coefficient, plasma zinc levels had an inverse correlation with the duration of ulcer in patients with chronic leg ulcers - in that lower zinc levels were observed in patients with longer duration of ulcers.

However, there was no correlation between zinc levels and albumin and protein levels. This is in contrast to study in which albumin deficient recruits had a two and half greater risk of being zinc deficient compared to those with adequate serum albumin levels^25^. There was no correlation between zinc levels and albumin and protein levels in this study can be explained by the fact that there was no significant difference between albumin and protein levels in patients with chronic leg ulcers (who had significantly lower plasma zinc levels) and controls.

Using multivariate analysis, zinc levels were considerably lower in low socio-economic groups compared to the middle socio-economic groups. This is comparable with a study in which a Persian population was sampled^26^. Anthropometric measurements, serum zinc and copper analysis and socio-economic status were determined. Urban residents had higher serum zinc (*P*<0.01) than rural residents. Similarly, there was significantly higher serum zinc levels in the literate subgroup as compared to the illiterate subgroup.

## CONCLUSION

The mean plasma zinc was significantly lower (*P*<0.01) in patients with chronic leg ulcers (92.808±16.689 µg/dl) compared with the controls (109.413±9.304 µg/dl). The difference in the mean albumin and protein levels of the patients with chronic leg ulcers and the controls was not statistically significant. Plasma zinc levels had an inverse correlation with the duration of ulcer in patients with chronic leg ulcers. Further studies need to be carried out to determine the role of zinc supplementation in facilitating healing in patients with chronic leg ulcers in Nigeria.
